# Iatrogenic Botulism Following Cosmetic Botulinum Toxin Injection Presenting With Respiratory Distress: A Case Report

**DOI:** 10.7759/cureus.98836

**Published:** 2025-12-09

**Authors:** Haider Zahur, Jawad Malik, Laraib Arshad

**Affiliations:** 1 Intensive Care, University Hospitals of Leicester NHS Trust, Leicester, GBR; 2 Respiratory Medicine, University Hospitals of Leicester NHS Trust, Leicester, GBR

**Keywords:** aesthetic medicine, cosmetic injections, iatrogenic botulism, respiratory distress, toxin-induced respiratory failure

## Abstract

Botulinum toxin (BoNT) is extensively used across a wide range of therapeutic and cosmetic applications due to its efficacy in inhibiting neuromuscular transmission and its well-established safety profile. While adverse effects are typically localized and transient, systemic complications - particularly iatrogenic botulism - are rare but potentially life-threatening events. Iatrogenic botulism results from unintended systemic spread of the neurotoxin, which can occur even at doses commonly used for cosmetic purposes. We report a rare and severe case of iatrogenic botulism in a previously healthy 47-year-old male who developed progressive neurological symptoms following cosmetic BoNT injections to the glabellar region. Within days of the procedure, the patient experienced worsening bilateral ptosis, ophthalmoplegia, dysarthria, and dysphagia, which progressed to respiratory muscle involvement requiring mechanical ventilation in an intensive care setting. Neurophysiological studies demonstrated findings consistent with a presynaptic neuromuscular transmission defect. The case presented several diagnostic challenges, as the early clinical features mimicked other neuromuscular disorders such as myasthenia gravis or brainstem stroke. Despite this, prompt recognition based on clinical suspicion led to the timely administration of heptavalent botulinum antitoxin, which was followed by gradual neurological improvement. This case underscores the importance of early identification and management of systemic BoNT toxicity, even in the context of routine cosmetic use. It also highlights the critical need for clinician awareness, early referral to specialized care, and a high index of suspicion in patients presenting with acute cranial neuropathies following BoNT administration. This report aims to highlight the potential for severe systemic complications associated with BoNT use and to emphasize the importance of early antitoxin administration.

## Introduction

Botulinum toxin (BoNT) is a potent neurotoxin produced by *Clostridium botulinum*, which acts by cleaving soluble N-ethylmaleimide-sensitive factor attachment protein receptor (SNARE) proteins necessary for acetylcholine release at the presynaptic terminals of neuromuscular junctions, ultimately resulting in flaccid paralysis of the affected muscles [1]. Initially recognized for its paralytic effects in botulism, BoNT has since been repurposed for a wide array of clinical applications. Its use has expanded significantly over the past two decades and now encompasses treatment for dystonias, spasticity, chronic migraine, hyperhidrosis, and various urological and gastrointestinal disorders, in addition to its widespread popularity in aesthetic medicine [1-3].

Among the seven BoNT serotypes (A-G), serotypes A and B are most commonly used in clinical settings, with BoNT-A formulations -such as onabotulinumtoxinA (Botox®), abobotulinumtoxinA (Dysport®), and incobotulinumtoxinA (Xeomin®) - being the predominant agents used in cosmetic procedures. These formulations are generally considered safe when administered at recommended doses by trained professionals, with adverse effects usually limited to mild, local reactions such as bruising, ptosis, or asymmetry.

However, iatrogenic botulism is a rare but serious adverse event arising from the inadvertent systemic spread of the toxin beyond the intended injection site. The true incidence is difficult to ascertain due to underreporting, non-specific early symptoms, and misdiagnosis, but several cases have recently drawn global attention to this emerging complication [4,5].

Systemic botulism classically presents with a descending, symmetrical paralysis, beginning with cranial nerve involvement - manifesting as diplopia, dysarthria, dysphonia, and dysphagia - progressing to limb weakness and, in severe cases, respiratory failure requiring ventilatory support [6]. The clinical overlap with other neuromuscular conditions, such as myasthenia gravis, Guillain-Barré syndrome, and brainstem stroke, further complicates timely diagnosis. Electrophysiological studies and toxin assays, though helpful, are often delayed or unavailable, making early clinical recognition essential [6].

Management of iatrogenic botulism hinges on rapid diagnosis and the prompt administration of heptavalent botulinum antitoxin (HBAT), which neutralizes circulating toxin but does not reverse established neuromuscular blockade. Delay in antitoxin administration has been associated with worse outcomes and prolonged recovery. Supportive care, including respiratory monitoring and rehabilitation, plays a central role in recovery, which can take weeks to months depending on severity.

In this report, we present a rare case of iatrogenic botulism following cosmetic BoNT injection in a previously healthy individual. The case illustrates the diagnostic uncertainty, management complexities, and need for heightened clinician awareness in recognizing this potentially life-threatening but treatable condition.

## Case presentation

A 47-year-old previously healthy male presented to the Emergency Department (ED) with a five-day history of progressively worsening diplopia, dysphagia, hoarseness, and shortness of breath. He reported having undergone a cosmetic botulinum toxin type A (BoNT-A) injection to the forehead for wrinkle reduction five days prior to the onset of symptoms. There was no history of recent illness, gastrointestinal symptoms, trauma, or travel, and he was not on any regular medications.

Upon arrival, the patient was alert but in severe respiratory distress. He exhibited audible stridor and hoarseness and was unable to lift his head due to profound weakness in the neck flexors. Vital signs revealed sinus tachycardia with a heart rate of 120-130 beats per minute, a respiratory rate of 35 breaths per minute, and blood pressure of 110/80 mmHg. His oxygen saturation was 98% on 15 L/min oxygen via a non-rebreather mask, indicating significant respiratory compromise (Table 1).

**Table 1:** Vital signs on presentation Table 1 summarizes the vital signs of the 47-year-old male patient upon arrival at the Emergency Department. Despite significant respiratory distress, the patient remained hemodynamically stable, with oxygen saturation maintained at 98% on 15L oxygen via a non-rebreather mask.

Vital Sign	Measurement
Heart Rate	120-130 beats per minute (sinus tachycardia)
Blood Pressure	110/80 mmHg
Respiratory Rate	35/min
Oxygen Saturation (SpO₂)	98% on 15 L/min via a non-rebreather mask
Temperature	37
Level of Consciousness	Alert

Neurological examination revealed severe weakness of the neck flexors (Medical Research Council (MRC) grade 1/5), generalized hypotonia, and flaccid weakness with greater proximal than distal involvement in all four limbs. Proximal strength in the upper limbs was graded at 2/5 and distal at 3/5, while the lower limbs were 3/5 proximally and 4/5 distally. Deep tendon reflexes were diminished throughout. The patient also had bilateral ptosis, diplopia, and signs of bulbar involvement, including dysphagia and hoarseness, suggesting cranial nerve dysfunction (Table 2).

**Table 2:** Neurological assessment on admission Table 2 summarizes the neurological findings on admission. Notable signs included severe neck flexor weakness, generalized hypotonia, and diminished deep tendon reflexes, with bilateral ptosis and bulbar palsy indicative of systemic botulism.

Neurological Feature	Findings
Neck Flexor Strength	Severe weakness, MRC grade 1/5
Muscle Tone	Generalized hypotonia
Muscle Weakness	Flaccid weakness, proximal > distal involvement
Upper Limbs Strength (Proximal)	02 May
Upper Limbs Strength (Distal)	03 May
Lower Limbs Strength (Proximal)	03 May
Lower Limbs Strength (Distal)	04 May
Deep Tendon Reflexes	Diminished throughout the upper and lower limbs
Cranial Nerves	Bilateral ptosis, diplopia, bulbar palsy (dysphagia, hoarseness)
Clinical Impression	Neuromuscular junction disorder; suspicion of iatrogenic botulism post cosmetic BoNT injection

**Figure 1:**
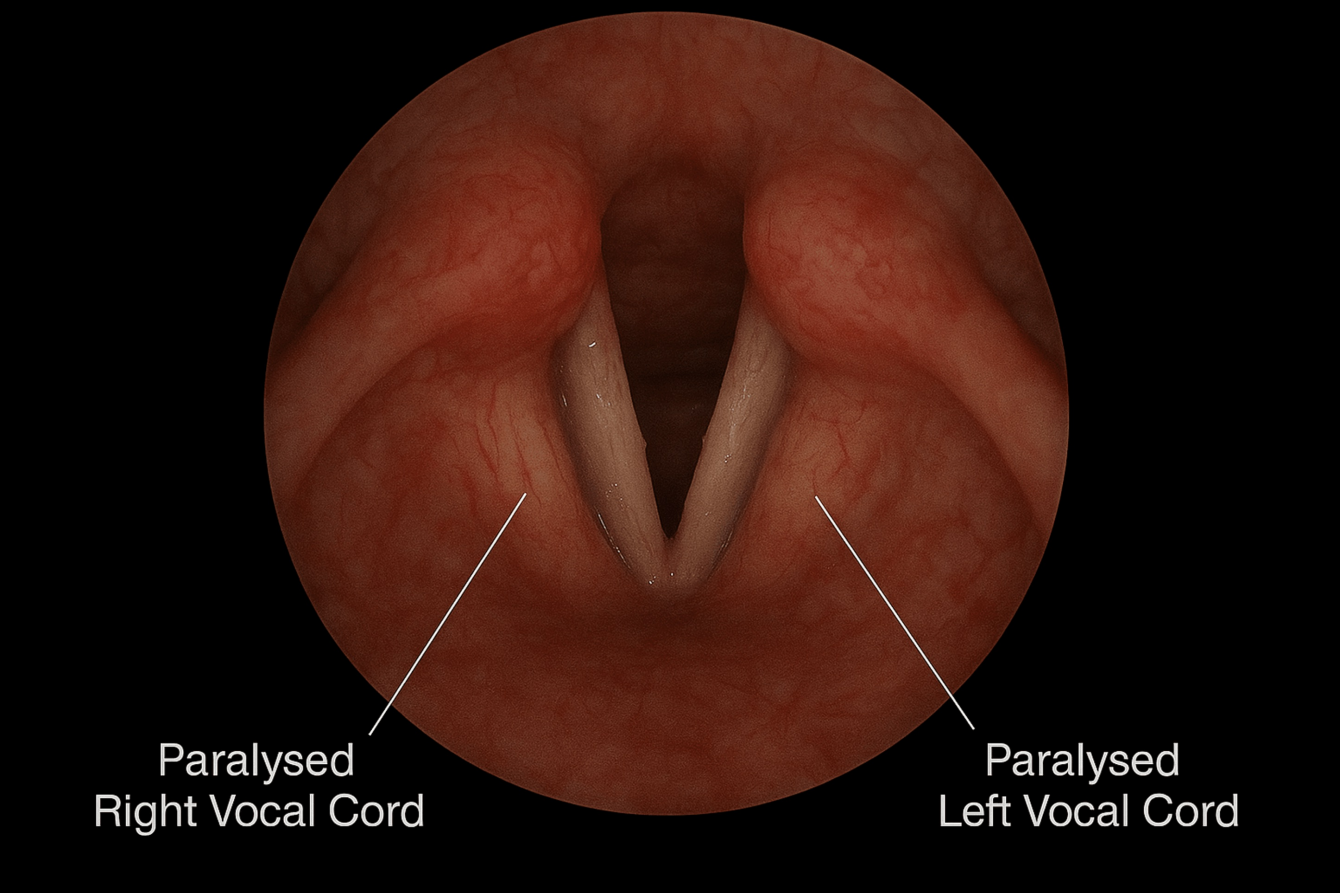
Flexible nasoendoscopic view demonstrating bilateral vocal cord palsy Flexible nasoendoscopic examination showing bilateral vocal cord palsy. The white arrows indicate the immobile vocal cords, which are positioned in the paramedian position, resulting in a narrow glottic gap. The epiglottis and supraglottic structures appear normal, with no evidence of supraglottitis or anatomical abnormality. This endoscopic view highlights the characteristic appearance of vocal cord immobility in bilateral palsy

Flexible naso-endoscopy done by ENT demonstrated bilateral vocal cord palsy with normal laryngeal anatomy and no evidence of supraglottitis (Figure 1).

Arterial blood gas on 15 L of oxygen showed type 1 respiratory failure (Table 3).

**Table 3:** Arterial blood gas (ABG) analysis on admission on 15 L oxygen via a non-rebreather mask Analysis shows type 1 respiratory failure and inadequate oxygenation despite the administered oxygen.

Parameter	Result	Reference Range	Interpretation
pH	7.37	7.35-7.45	Normal
PaO₂	7.7 kPa	10.7-13.3 kPa	Hypoxaemia despite 15 L oxygen
PaCO₂	5.1 kPa	4.7-6.0 kPa	Normal
Oxygen Saturation (SpO₂)	98%	95-100%	Maintained on 15 L/min oxygen

Due to progressive respiratory compromise and upper airway obstruction from bilateral vocal cord palsy, the patient was emergently intubated and admitted to the intensive care unit (ICU). Public Health authorities were notified of a suspected case of iatrogenic botulism. On day two of hospitalization, he received intravenous heptavalent botulinum antitoxin (20 mL), and serum samples were sent for confirmatory botulinum toxin assay.

A neurology consultation was obtained, and adjunctive treatment with pyridostigmine 30 mg three times daily was initiated. To rule out other potential autoimmune or infectious causes of his presentation, a comprehensive workup was performed. Tests for anti-nuclear antibodies (ANA), extractable nuclear antigen (ENA) panel, HIV, syphilis serology, and cryoglobulin levels were all negative (Table 4).

**Table 4:** Autoimmune and infectious screening results Comprehensive laboratory testing was performed to evaluate for underlying autoimmune or infectious causes of the patient’s presentation. All results were negative, effectively ruling out systemic autoimmune disease.

Test	Result	Interpretation
Anti-nuclear Antibodies (ANA)	Negative	No evidence of autoimmunity
Extractable Nuclear Antigen (ENA) Panel	Negative	No specific connective tissue disease markers
HIV Screening	Negative	No HIV infection detected
Syphilis Serology	Negative	No evidence of syphilis
Cryoglobulin Levels	Negative	No cryoglobulinaemia

On day three, electromyography and nerve conduction studies were done (Table 5).

**Table 5:** Neurophysiological studies supporting presynaptic neuromuscular junction disorder Neurophysiological testing revealed a pattern typical of botulinum toxin effect, including low resting CMAP amplitudes and a significant incremental response on high-frequency stimulation. These findings support a diagnosis of presynaptic neuromuscular junction dysfunction, consistent with iatrogenic botulism.

Test	Findings
Electromyography (EMG)	Low-amplitude compound muscle action potentials (CMAPs) at rest
Nerve Conduction Studies	Marked incremental response on high-frequency (20–50 Hz) repetitive nerve stimulation
Interpretation	Findings are consistent with a presynaptic neuromuscular junction disorder, such as botulism

A trial extubation was attempted but failed immediately due to persistent stridor and airway compromise, necessitating surgical tracheostomy on day four. Ventilator-associated pneumonia developed on day five and was treated with intravenous antibiotics, contributing to a prolonged tracheostomy weaning period.

By day 14, gradual neurological improvement was observed with increased proximal and distal limb strength and partial recovery of ptosis (Table 6).

**Table 6:** Neurological status on admission and after two weeks of supportive care This table compares the patient's neurological findings at admission and after 14 days of supportive management. While there was marked improvement in both proximal and distal limb strength, full recovery had not yet been achieved. Mild residual ptosis and proximal weakness persisted, consistent with the expected gradual resolution of iatrogenic botulism.

Neurological Parameter	Day 1 (Admission)	Day 14	Interpretation
Neck Flexor Strength (MRC)	01-May	04-May	Improved but not fully recovered
Upper Limb Strength – Proximal	02-May	03-May	Moderate improvement
Upper Limb Strength – Distal	03-May	04-May	Improved but residual weakness
Lower Limb Strength – Proximal	03-May	04-May	Improved but residual weakness
Lower Limb Strength – Distal	04-May	05-May	Full recovery
Ptosis	Bilateral, complete	Mild residual	Partial resolution

The patient was transferred to the neurology ward for continued respiratory support and rehabilitation. Serum samples were submitted to the national reference laboratory for botulinum toxin assay. Results remained pending at the time of manuscript preparation, and the neurology team continued to monitor the case for confirmatory data.

## Discussion

Iatrogenic botulism is an uncommon but increasingly recognized complication of BoNT use, arising in both therapeutic and cosmetic contexts. Traditionally, botulism has been classified into foodborne, wound, and infant forms; however, with the widespread availability of BoNT for aesthetic procedures, iatrogenic botulism has emerged as a distinct clinical entity. Contributing factors include inappropriate dosing, off-label use, unlicensed formulations, and administration by untrained personnel [7].

Recent reports have highlighted a global rise in cases related to cosmetic procedures. A large retrospective study of illegal cosmetic injections found that nearly half of affected patients had received BoNT from unverified sources, often in non-medical environments, with moderate-to-severe presentations in over 60% of cases [7]. Similarly, several reports from Asia and Europe have described cases of iatrogenic botulism following administration of counterfeit or high-dose products, underscoring the importance of proper dosing, product authenticity, and clinician expertise [8].

Clinically, iatrogenic botulism is indistinguishable from other forms of botulism, manifesting as a descending, symmetrical flaccid paralysis that typically begins with cranial nerve involvement - ptosis, diplopia, dysarthria, and dysphagia - followed by limb and respiratory muscle weakness [6]. The incubation period generally ranges from a few days to one week, depending on the dose and injection site [9]. In our case, the onset of these hallmark neurological features within days of cosmetic BoNT injection was pivotal in establishing the diagnosis.

Electrophysiological testing plays a crucial role in confirming the diagnosis and differentiating it from other neuromuscular disorders such as myasthenia gravis or Guillain-Barré syndrome. Typical findings include low compound muscle action potential (CMAP) amplitudes with an incremental response on high-frequency repetitive nerve stimulation - features consistent with a presynaptic neuromuscular junction blockade. These findings were demonstrated in our patient and corroborated the clinical diagnosis.

Management of iatrogenic botulism requires early clinical suspicion, prompt administration of heptavalent botulinum antitoxin (HBAT), and supportive care, including vigilant respiratory monitoring. The Centers for Disease Control and Prevention (CDC) recommends administration of antitoxin as soon as botulism is suspected, as it neutralizes only circulating toxin and does not reverse internalized toxin effects. Recovery depends on the regeneration of new synaptic terminals, a process that may take weeks to months. Our patient required prolonged ventilatory support despite timely antitoxin, reflecting the severity of neuromuscular involvement and the recovery course typical of iatrogenic botulism.

From a public health standpoint, this case underscores the growing concern regarding counterfeit and unregulated BoNT preparations. Strengthening regulatory oversight, enforcing product authentication, and enhancing clinician education are essential preventive measures. Clinicians should maintain a high index of suspicion for iatrogenic botulism in patients presenting with descending paralysis and recent cosmetic procedures, as timely recognition and management are key to minimizing morbidity and mortality.

## Conclusions

Clinicians should maintain a high index of suspicion for botulism in patients presenting with progressive cranial nerve deficits and respiratory symptoms following cosmetic BoNT injections. Timely diagnosis, supported by neurophysiological studies, is crucial for distinguishing botulism from other potential causes of neuromuscular paralysis. Early administration of botulinum antitoxin, along with intensive supportive care, including airway protection and ventilatory support, is essential to prevent further progression of the disease and minimize both morbidity and mortality.

This case highlights the rare yet serious risk of iatrogenic botulism associated with cosmetic BoNT use and underscores the need for awareness among clinicians in recognizing and managing such complications effectively.
